# Phase-Selective Corrosion Kinetics of Marine Copper Alloys Under High-Speed Dynamic Seawater Erosion

**DOI:** 10.3390/ma19204381

**Published:** 2026-10-19

**Authors:** Xuelong Hao, Jiangda Tang, Yongjun Tian, Jingtong Zhang, Shuhui Huang, Lei Fan

**Affiliations:** 1 National Center of Analysis and Testing for Nonferrous Metals and Electronic Materials, China GRINM Group Co., Ltd., Beijing 100088, China; tangjiangda@163.com (J.T.);; 2 Guobiao (Beijing) Testing & Certification Co., Ltd., Beijing 101407, China; 3 Fastener Group (Guangdong) Co., Ltd., Guangzhou 215155, China; 4 State Key Laboratory of Nonferrous Structural Materials, China GRINM Group Co., Ltd., Beijing 100088, China; huangshuhui@grinm.com; 5 GRIMAT Engineering Institute Co., Ltd., Beijing 101407, China; 6 School of Mechanical and Electrical Engineering, China University of Mining and Technology-Beijing, Beijing 100083, China; flcumtb@163.com; 7 Key Laboratory of Intelligent Mining and Robotics, Ministry of Emergency Management, Beijing 100083, China

**Keywords:** marine copper alloy, simulation of the ocean, erosion–corrosion, microscopic damage

## Abstract

**Highlights:**

- A rotating erosion–corrosion rig reproduces high-speed flowing seawater service conditions.
- The α phase is preferentially corroded, driving damage from interface cracks to ≈100 μm pits.
- Peak corrosion rate occurs at 24 h, when the corrosion product film is unstable and spallable.
- Prolonged erosion–corrosion builds a stable protective layer that lowers the corrosion rate.
- The findings support material selection and life prediction for marine propeller components.

**Abstract:**

Nickel–aluminum bronze is a key structural material for marine propellers, yet its corrosion behavior under dynamic seawater erosion remains inadequately elucidated. To clarify the corrosion kinetics under high-speed dynamic seawater erosion of the ZCuAl10-4-4-2 marine copper alloy, erosion–corrosion tests and multi-method characterizations were conducted. The results illustrate that the α phase exhibited a higher corrosion susceptibility, driving micro-damage evolution: κ/α interface crack initiation at 4 h, significant crack propagation with a dense κ_II_ phase at 24 h, and pit formation (≈100 μm) at 48 h. Dynamic erosion induced critical electrochemical changes at 4 h and 24 h, with reduced charge transfer resistance, lower corrosion potential (*E*_corr_) and elevated current density; the 24 h stage showed the peak corrosion rate due to unstable, spallable corrosion product films. Prolonged erosion–corrosion promoted the formation of a stable protective corrosion product layer, which enhanced the alloy’s corrosion resistance and reduced its corrosion rate.

## 1. Introduction

Nickel–aluminum bronze (NAB), a high-performance copper-based alloy, is widely used in key engineering fields such as marine engineering, automotive manufacturing, and aerospace due to its high strength, excellent corrosion resistance, and superior casting and processing properties. Particularly in the marine sector, NAB is a critical material for manufacturing large-scale propellers, pumps, valves, and fluid handling components [1,2,3,4,5]. During service, these components are continuously exposed to seawater and hydrodynamic flow, where elevated flow velocities can intensify surface shear, accelerate mass transport, and destabilize corrosion-product films formed on the alloy surface [6]. Such coupled effects can substantially alter the corrosion kinetics and surface damage evolution of NAB, thereby affecting its long-term structural integrity and service reliability. Therefore, understanding the degradation behavior and underlying corrosion mechanisms of NAB under dynamic seawater conditions is essential for evaluating its durability and predicting the service lifetime of marine components.

The corrosion behavior of NAB is strongly influenced by its complex multiphase microstructure and the distinct electrochemical activities of its constituent phases. Previous studies have shown that the corrosion response of NAB varies considerably with the surrounding environment. For instance, the corrosion kinetics and mechanisms in seawater of NAB were systematically evaluated by Song et al. [7] through static immersion and electrochemical testing in acidic and neutral 3.5 wt.% NaCl solutions. The static corrosion study found that in acidic NaCl solution, the κ phases and small-sized phases within β′ corroded preferentially, while in a neutral NaCl solution, the aluminum-rich κ phases and small-sized phases within β′ were protected by the formation of an Al_2_O_3_ protective film, while the copper-rich α phase corroded preferentially. Similar protective-film formation has also been reported for other copper-based marine alloys. For instance, a Cu2O-rich passive film formed on Cu-Ni alloys during marine exposure was reported to exhibit strong adhesion to the substrate and effectively suppress further corrosion [8]. Although these studies have provided important insights into the phase-dependent corrosion behavior and surface-film protection of copper-based alloys under static conditions, the stability and protective function of such surface films under dynamic seawater flow remain less well understood.

Recent studies have increasingly focused on the erosion–corrosion behavior of copper-based alloys under dynamic seawater conditions. For example, the erosion–corrosion performance of copper–nickel alloys with different compositions and preparation processes under dynamic seawater environments was studied by Chu et al. [9]. It was found through research that the addition of chromium to copper–nickel alloys can induce the formation of the (Ni/Fe)_3_Cr phase, which enhances the mechanical strength of the alloy and improves the erosion–corrosion resistance of copper–nickel alloys, revealing the regulation of alloy composition on dynamic corrosion performance. Similarly, Wang et al. [10] investigated the microstructural evolution of NAB under erosion–corrosion at the flow rate of 3–5 m/s. A multilayer corrosion-product film was formed on the alloy surface after pre-immersion in seawater, consisting of a relatively loose CuO layer, a more compact Cu_2_O layer, and a dense inner layer containing Cu_2_O and Al_2_O_3_. This film structure contributed to suppressing the subsequent erosion–corrosion damage. A protective corrosion-product film was also observed by Cao et al. [11] on B10 copper–nickel alloys under the erosion–corrosion of sandy seawater conditions. It is confirmed that, under a scour rate of 0–1.5 m/s, the existence of a protective film could significantly reduce the corrosion rate. More recently, Li et al. [12] demonstrated that seawater flow velocity strongly affected the evolution and protectiveness of the corrosion-product film. With increasing flow velocity, the damage to the corrosion-product film became progressively more pronounced. The film could effectively protect the alloy surface under relatively low flow velocities in clean seawater, whereas its protective function was substantially weakened in sand-containing seawater. These studies indicate that hydrodynamic conditions play a critical role in regulating the formation, stability, and protective function of corrosion-product films on copper-based alloys. However, existing studies have predominantly focused on static or relatively low-velocity flow conditions, and the corrosion and surface-damage behavior of NAB under sustained dynamic seawater flow remains insufficiently understood. In particular, the coupled effects of hydrodynamic shear, corrosion-product-film evolution, and the heterogeneous microstructure of NAB have yet to be systematically clarified.

Herein, a fully immersed multi-factor coupled rotating erosion–corrosion test was employed to investigate the degradation behavior of a high-strength NAB alloy under dynamic artificial seawater conditions. The evolution of surface damage was systematically examined as a function of exposure time at a controlled linear velocity of 5 m/s. By combining corrosion weight loss analysis, macro- and micro-morphological observation, as well as electrochemical impedance spectroscopy (EIS) and polarization curve measurements, this study elucidates the corrosion kinetic behavior, erosion–corrosion synergistic mechanisms, and surface damage characteristics of this alloy in dynamic seawater environments. The results provide experimental evidence and theoretical guidance that more closely align with actual service conditions for the development, material selection, and lifetime prediction of high-performance marine propeller materials.

## 2. Materials and Methods

### 2.1. Materials

The copper alloy ZCuAl10Fe4Ni4Mn2 was used in this work, and is hereafter designated as ZCuAl10-4-4-2. Table 1 shows the main chemical composition of common nickel–aluminum bronzes, and Table 2 shows the chemical composition of the ZCuAl10-4-4-2 copper alloy.

### 2.2. Sample Preparation

The nickel–aluminum bronze specimen was precisely processed into pieces with a size of 30 mm × 15 mm × 3 mm by wire cutting technology. Before the test, the working surfaces were sequentially ground using 240-, 500-, 1000- and 2400-grit silicon carbide sandpaper, and then treated with a diamond polishing paste with a particle size of 3 μm to obtain a scratch-free mirror polish. The samples were then washed with deionized water and ultrasonically cleaned in ethanol for 20 min before drying and storage.

### 2.3. Artificial Seawater Erosion–Corrosion

**Figure 1:**
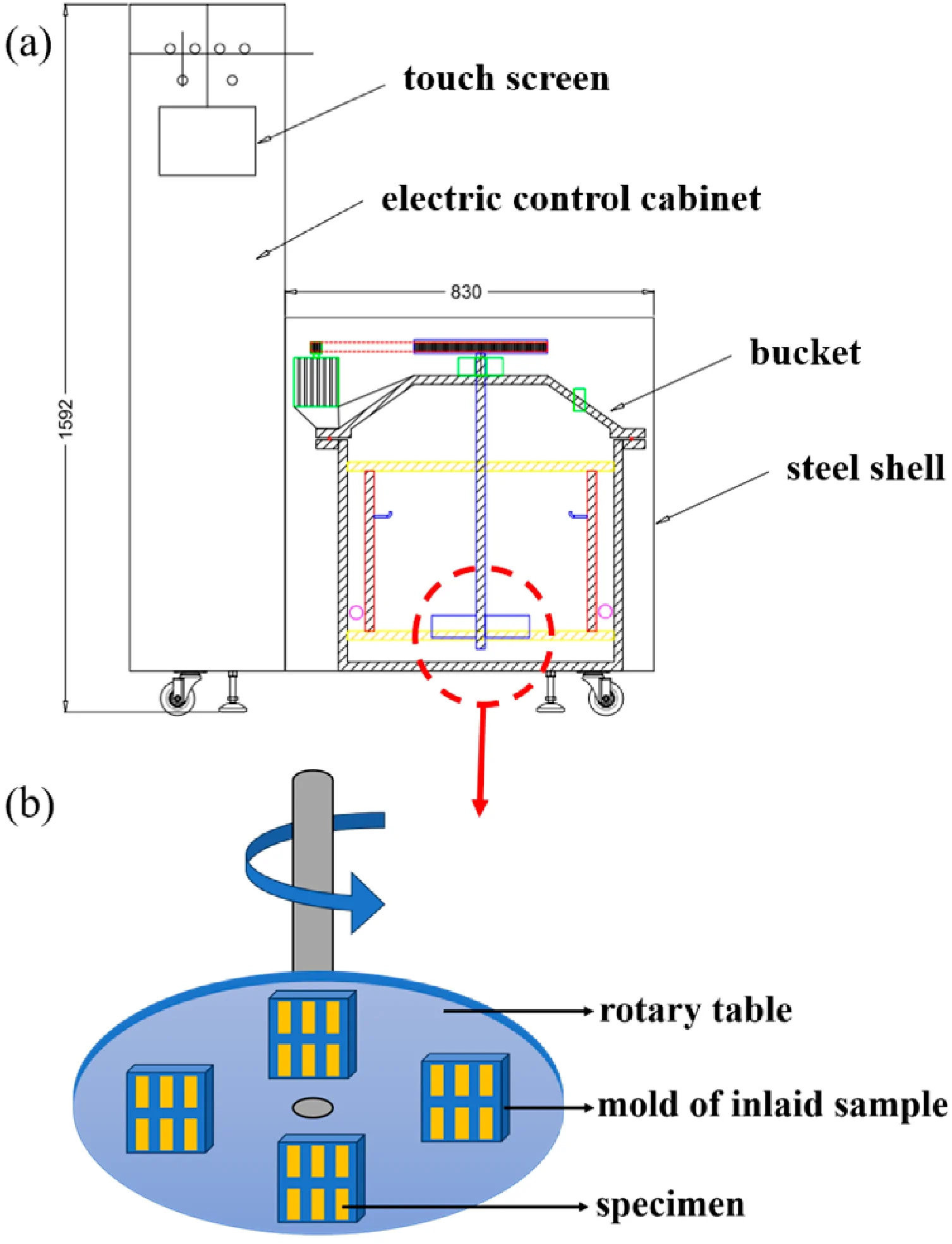
Schematic diagram of the copper alloy corrosion–erosion testing machine. (**a**) A cross-sectional view; (**b**) rotating sample plate.

The rotating disk method erosion test was carried out using the copper alloy corrosion erosion test instrument (China United Test & Certification Co., Ltd., Beijing, China (Figure 1)). The corrosive medium is artificial seawater (Table 3). The linear velocity of the sample end was 5 m/s. The scour angle was 0°; the test times were 0 h, 2 h, 4 h, 6 h, 12 h, 24 h, 48 h, 72 h and 96 h respectively. The test temperature was room temperature. The samples were electrochemically tested immediately after the erosion–corrosion test.

The international standard for the formulation of artificial seawater isASTM D1141 [13]. The composition of artificial seawater is shown in Table 3.

### 2.4. Microstructure Characterization

The microstructure, elemental distribution and corrosion morphology of the samples at different exposure times were analyzed by a scanning electron microscope (SEM, JSM-IT800, JEOL, Tokyo, Japan), and the analysis of micro-area elements was conducted by an energy-dispersive spectrometer (EDS, XPLORE65, OXFORD INSTRUMENTS, Oxford, UK). 

### 2.5. Electrochemical Test

The electrochemical comprehensive tester (PARSTAT-4000A, AMETEK, Oak Ridge, TN, USA) was used to conduct the three-electrode system with the Ag-AgCl reference electrode and platinum counter electrode. The test medium was 3.5 wt.% NaCl solution; the exposed area of the sample was 1 cm^2^; the test temperature was room temperature; the electrochemical impedance frequency test range was 10^−2^–10^5^ Hz; the amplitude was 10 mV; and the polarization curve test range was open circuit potential (OCP) ± 0.25 V. The electrochemical impedance data were simulated by ZView2 software. The open-circuit potential (OCP) stabilization time was 1 h, and the polarization scan rate was 0.167 mV/s.

### 2.6. Corrosion Weight Loss Test

The erosion–corrosion rate of the sample was evaluated by the corrosion weight loss test. According to the requirements of ASTM G1-03 [14], 500 mL of both concentrated hydrochloric acid and distilled water were prepared and added to 1000 mL of solution as the cleaning solution. The test surface of the sample after erosion–corrosion was immersed in the cleaning solution under ultrasonic vibration for 3 min, and the test surface was washed with anhydrous ethanol and dried in the oven. The corrosion rate was calculated according to Equation (1) [15]:

Corrosion rate (μm/a) = 8.76 × 10^7^ × W/(A × T × D) (1)

In the equation, 8.76 × 10^7^ is a constant; W is the mass loss (g) before and after cleaning; A is the total test area (cm^2^) of the sample, 3 cm × 1.5 cm; T is the corrosion time (h); and D is the sample density (g·cm^−3^).

### 2.7. Scanning Kelvin Probe Force Microscopy (SKPFM) Test

Scanning Kelvin Probe Force Microscopy (SKPFM, Dimension ICON, Bruker, Santa Barbara, CA, USA) was utilized to characterize the Volta potential differences between distinct phases of the copper alloy specimen.

## 3. Results

### 3.1. Microstructures of ZCuAl10-4-4-2 Copper Alloy

**Figure 2:**
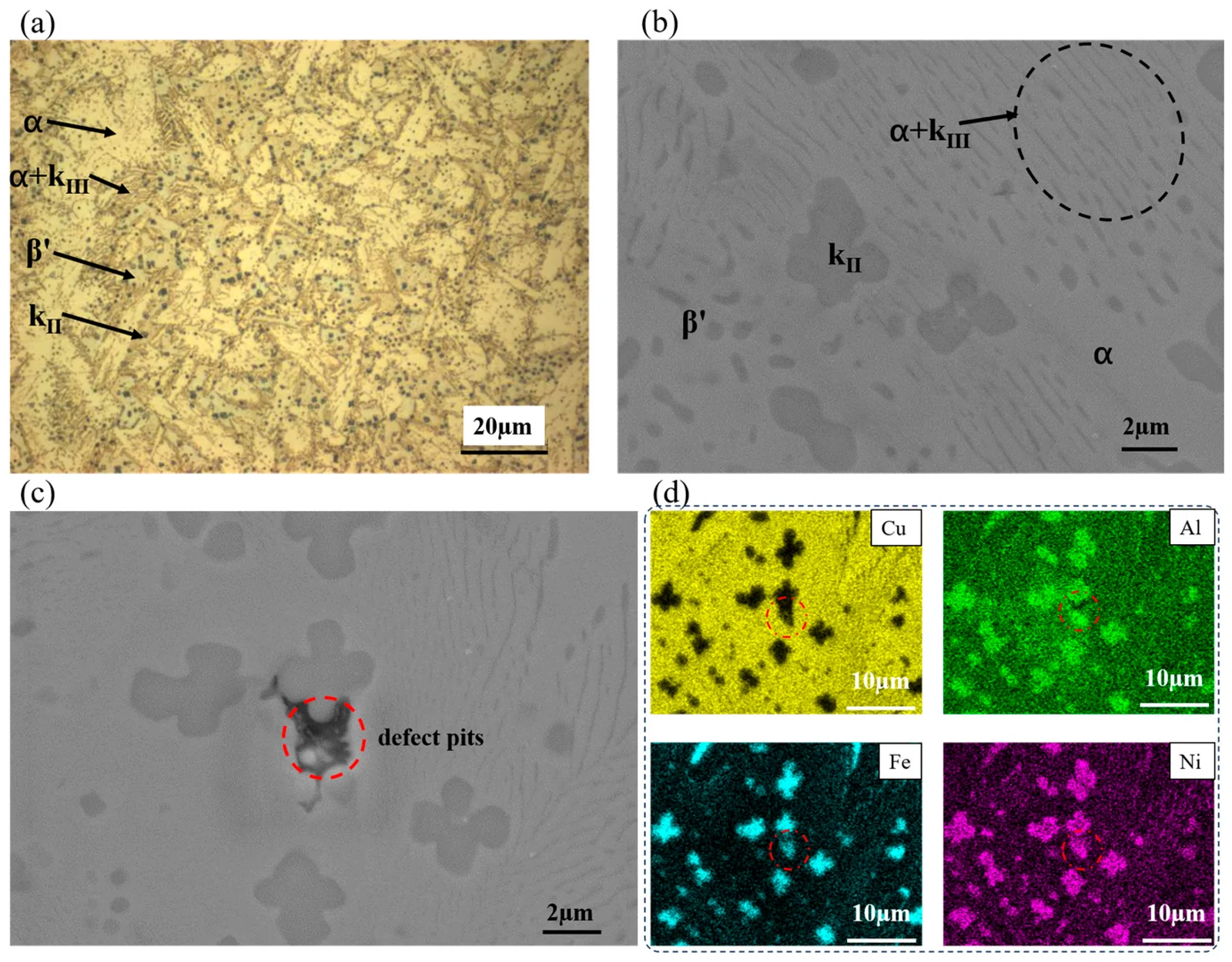
The microstructure of ZCuAl10-4-4-2 copper alloy. (**a**) Metallographic image; (**b**,**c**) SEM images; (**d**) EDS elemental mapping.

The surface of the ZCuAl10-4-4-2 copper alloy sample was ground and polished to remove surface oxides, and its microstructural morphology was examined at different magnifications. Figure 2b displays the microstructural morphology of the ZCuAl10-4-4-2 copper alloy, which is predominantly composed of spherical, petaloid, and lath-shaped κ precipitates, with the α matrix phase distributed intermittently. The κ precipitates originate from the high-temperature eutectoid decomposition of the β phase [16], and mainly consist of the κ_II_ phase (featuring spherical, petaloid or lath-shaped morphologies) and the κ_III_ phase with an interconnected network or lath-like structure. The elemental composition of each constituent phase in the ZCuAl10-4-4-2 copper alloy is listed in Table 3. Based on the data in Table 3 combined with Figure 2a,b, it can be inferred that the κ_II_ phase (dominated by Fe3Al) is primarily distributed at α-grain boundaries or α/β′-phase interfaces with a size range of 3–6 μm, whereas the κ_III_ phase (mainly composed of NiAl) exhibits a lath-like morphology along the α/β′-phase interfaces.

The β′ phase is primarily formed via the diffusionless martensitic transformation of the high-temperature β phase during rapid cooling. When nickel–aluminum bronze is subjected to solution treatment at 850–1000 °C, the body-centered cubic (BCC) β phase in the initial microstructure (composed of α and κ phases) remains thermally stable. Upon sufficiently rapid cooling, the β phase lacks adequate time to decompose into the α + κ eutectoid structure and instead transforms directly into the martensitic β′ phase through a shear-dominated mechanism. The β′ phase is susceptible to corrosion attack in NaCl solutions, shows an increased propensity for crack propagation in fatigue and stress corrosion tests, and thereby impairs the corrosion resistance of the copper alloy [17]. As illustrated in Figure 2a,b, the α and κ phases are extensively distributed and constitute the primary constituents of the alloy, while the β′ phase exists only in trace amounts. Table 4 presents the EDS-derived elemental composition of each phase in the ZCuAl10-4-4-2 copper alloy. Relative to the α phase, the κ phase possesses higher Fe, Al and Ni contents but a lower Cu concentration, resulting in its preferential dissolution in acidic environments.

**Figure 3:**
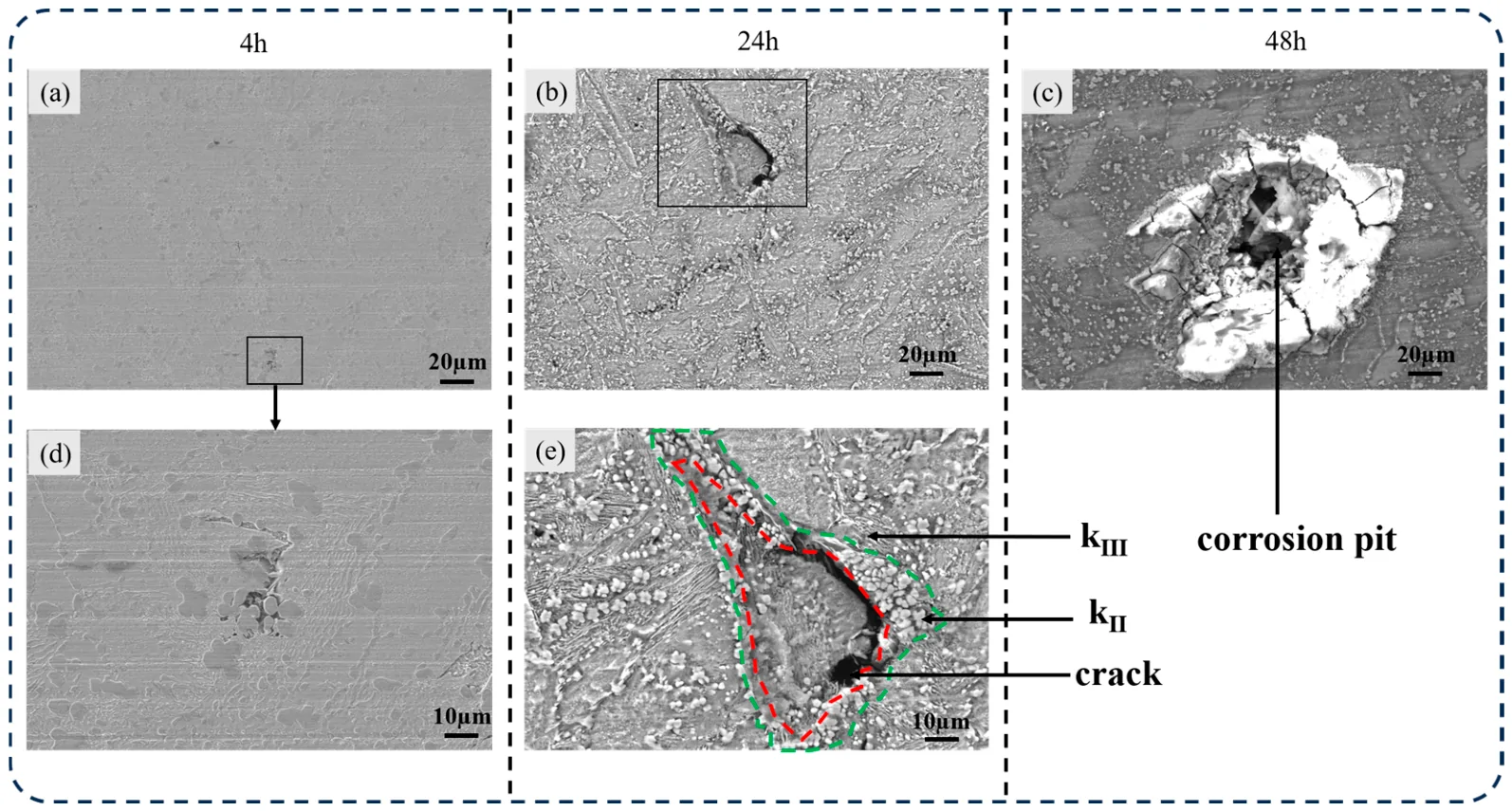
(**a**,**d**): Surface morphology of ZCuAl10-4-4-2 copper alloy after 4 h of erosion–corrosion test. (**d**) Shows a higher-magnification image of the region enclosed by the black box in (**a**); (**b**,**e**): Surface morphology of ZCuAl10-4-4-2 copper alloy after 24 h of erosion–corrosion test. (**e**) Shows a higher-magnification image of the region enclosed by the black box in (**b**); (**c**): Surface morphology of ZCuAl10-4-4-2 copper alloy after 48 h of erosion–corrosion test.

**Figure 4:**
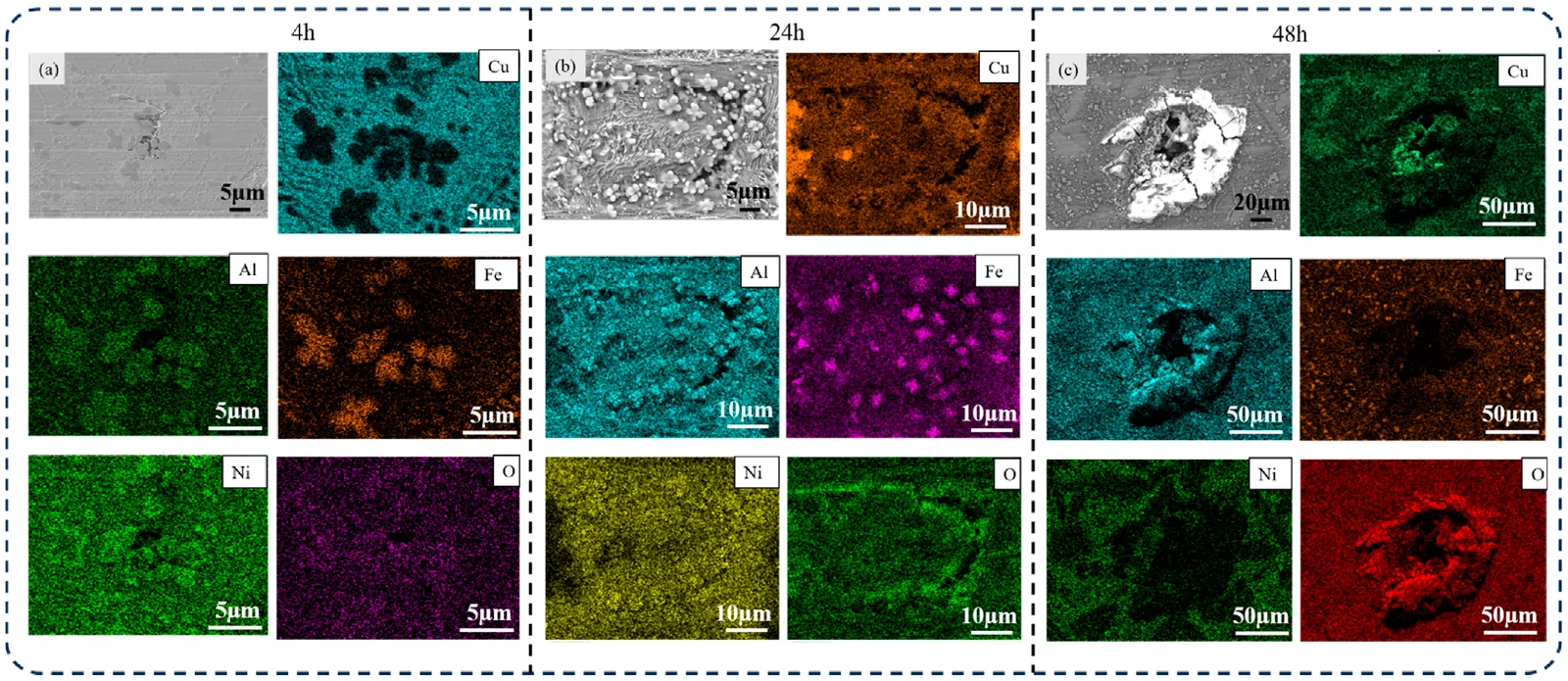
Energy-dispersive spectroscopy (EDS) of ZCuAl10-4-4-2 copper alloy after 4 h, 24 h and 48 h of erosion–corrosion test. (**a**) 4 h; (**b**) 24 h; (**c**) 48 h.

Petaloid defect pits were identified adjacent to the κ_II_ phase on the surface of the ZCuAl10-4-4-2 copper alloy (The red circle in Figure 2c,d). The elemental distribution of these defect pits is analogous to that of the κ_II_ phase, featuring elevated Fe and Ni contents and a reduced Cu concentration (Figure 2d). These defect pits, situated at the κ/α-phase boundaries, are pre-existing defect structures. Examination of the corrosion morphology of the ZCuAl10-4-4-2 copper alloy at various erosion–corrosion durations reveals three distinct evolutionary stages: crack initiation at 4 h, significant crack propagation at 24 h, and pit formation at 48 h. The surface morphologies of the ZCuAl10-4-4-2 copper alloy following 4 h, 24 h, and 48 h of erosion–corrosion are displayed in Figure 3. Following 4 h of erosion–corrosion, microcracks were initiated at the interfaces between the κ_II_ phase and adjacent phases; nevertheless, no oxygen enrichment was detected at these crack nucleation sites (Figure 4a). Following 24 h of erosion–corrosion, well-defined microcracks were observed on the specimen surface, whereas no corrosion pits were detected. In Figure 3e, the crack profile is delineated by the red dashed line, and the surrounding κ_II_ phase is marked by the green dashed line, signifying a relatively dense distribution of the κ_II_ phase in the vicinity of the microcracks. Elongated, ridge-shaped κ_III_ phases are distinctly observed in Figure 3e, which are generated by the preferential corrosion of the α phase in the α + κ_III_ structure. As shown in Figure 4b, a high density of κII phases was observed adjacent to the microcracks after 24 h of exposure, accompanied by pronounced enrichment of O and Al. This elemental distribution suggests the formation of Al- and O-rich products in these regions. Accordingly, during the initial stage of erosion–corrosion, Al- and O-rich products may preferentially form on the κ_II_ phase, and this may be associated with a lower dissolution rate of the κ_II_ phase than that of the α phase.

Distinct corrosion pits with a size of approximately 100 μm are detected on the copper alloy surface after 48 h of erosion–corrosion. As illustrated in Figure 3c and Figure 4c, the pits exhibited a crater-like morphology, characterized by raised edges and a depressed central region, and were surrounded by microcracks. In the central depressed region of these corrosion pits, the Al and O signals were stronger, the Ni and Fe signals were weaker, and the Cl signal was enhanced.

The ZCuAl10-4-4-2 copper alloy exhibits a microstructure composed of an α matrix, minor β′ martensite, and two types of κ precipitates—κ_II_ (Fe_3_Al-rich, spherical/petaloid/lath-shaped, 3–6 μm) and κ_III_ (NiAl-rich, lath-like)—with erosion-corrosion progressing through three stages: crack initiation at κ_II_/α interfaces after 4 h, crack propagation with Al-oxide formation on κ_II_ after 24 h, and the development of crater-like pits (~100 μm) enriched in Al, O, and Cl but depleted in Ni and Fe after 48 h.

### 3.2. Scanning Kelvin Probe Test Results 

**Figure 5:**
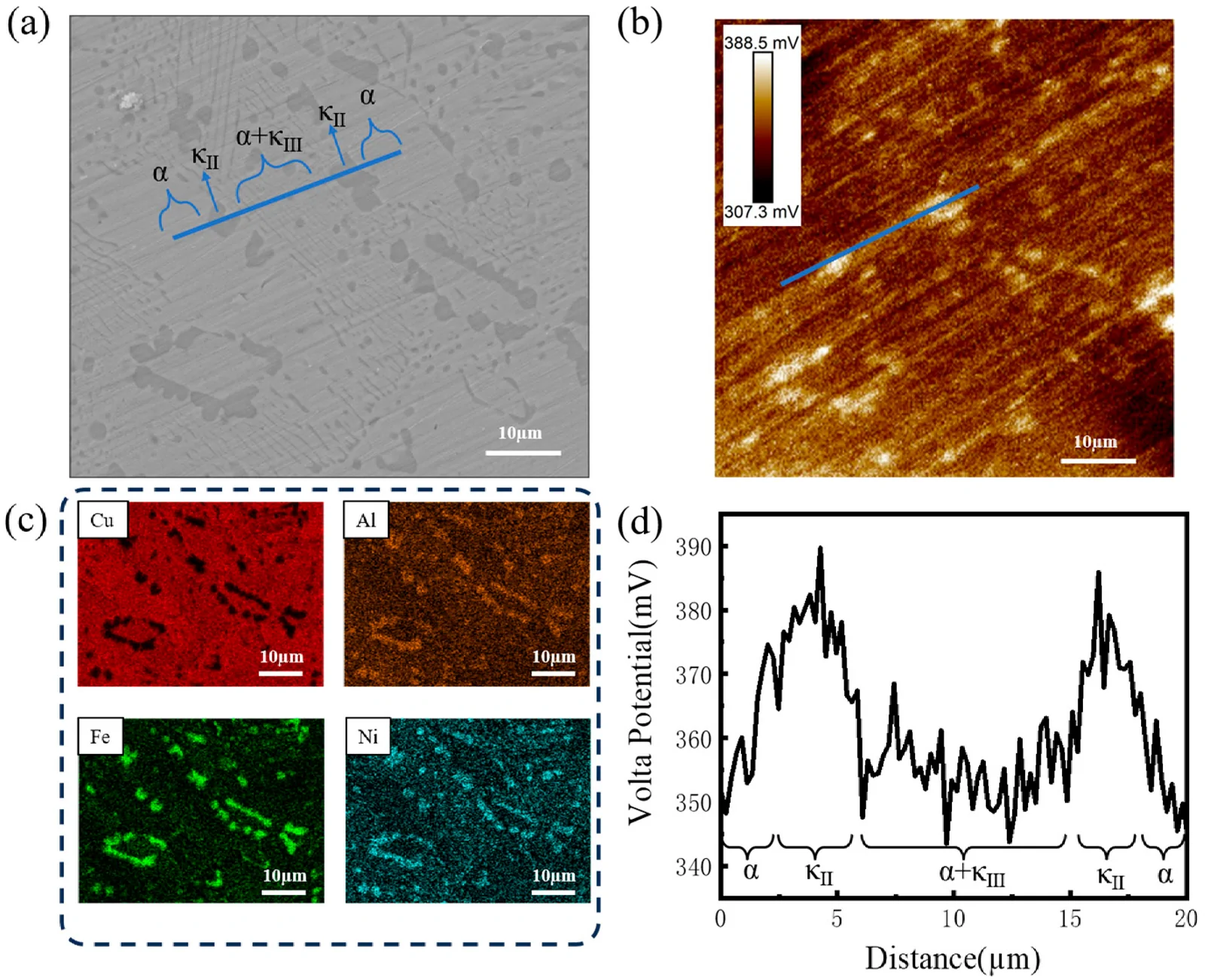
(**a**) A microstructural image; (**b**) a Volta potential map; (**c**) EDS results; and (**d**) the potential variation along the blue line marked in (**a**).

The Local Volta potential differences associated with the heterogeneous microstructure of the copper alloy were characterized using Scanning Kelvin Probe Force Microscopy (SKPFM). Absolute potential values are susceptible to multiple experimental parameters and necessitate rigorous measurement as well as precise instrument calibration. Accordingly, relative potential disparities between distinct constituent phases are predominantly investigated in the present work. Scanning Kelvin Probe Force Microscopy (SKPFM) measurements were performed, and the results are presented in Figure 5. A clear correspondence between the Volta potential distribution and microstructural characteristics can be observed by comparing Figure 5a,b. The elevated potential of the second-phase particles relative to the α matrix is identified in Figure 5d. The blue-marked line was examined sequentially through the α phase, κ_II_ phase, α + κ_III_ eutectoid structure, κ_II_ phase, and α phase. The corresponding Volta potential variations are plotted in Figure 5d. The maximum Volta potential difference between the κ_II_ and α phases is 58.3 mV, with the κ_II_ phase exhibiting a more positive potential than the α phase (Figure 5b,d).

In hydrochloric acid solution, Nakhaie et al. [17] measured the Volta potential values of individual constituent phases in nickel–aluminum bronze by SKPFM. They demonstrated that the α phase had a higher potential and acted as the cathode, while the κ phase served as the anode, resulting in intensified selective corrosion of κ in acidic environments. In contrast, in neutral 3.5 wt.% NaCl solution, the corrosion-product film formed on the κ phases exhibits a more noble Volta potential and lower electrical conductivity than that on the α phase. Consequently, the κ phases generally act as local cathodes, whereas the Cu-rich α phase acts as the anode and undergoes preferential dissolution, particularly near the α/κ_II_ interface. Ding et al. [18] examined the influence of microstructural features on the crack propagation behavior of copper alloys in 3.5 wt.% NaCl solution and reported the highest crack propagation rate in the α phase, further indicating the susceptibility of the α phase under neutral NaCl conditions. This neutral-environment behavior is supported by previous studies [19,20,21]. A protective Al_2_O_3_ film has been confirmed to form readily on the κ phase in neutral corrosive media, which suppresses the corrosion kinetics of κ [7]. Therefore, in neutral solutions, preferential dissolution occurs in the α phase at or near the α/κ boundaries, and the lath-like α + κIII structure exhibits enhanced corrosion susceptibility [22]. This opposite polarity indicates that the relative cathodic/anodic roles of the α and κ phases are strongly dependent on the testing environment.

In the ZCuAl10-4-4-2 alloy, the lower Volta potential of the α phase indicates its relatively more active electrochemical characteristics under seawater erosion–corrosion conditions. Consistent with the SKPFM results, crack initiation was preferentially observed at α/κII-phase interfaces, and these sites subsequently developed into localized corrosion damage with prolonged erosion–corrosion exposure.

### 3.3. Electrochemical Behavior of ZCuAl10-4-4-2 Copper Alloy After Corrosion Erosion

#### 3.3.1. EIS and Equivalent Circuit Fitting

Before applying equivalent-circuit models, the experimentally measured EIS responses were examined directly. The shape and size of the capacitive response in the Nyquist plots and the low-frequency impedance magnitude vary with erosion–corrosion exposure time. In the Bode phase-angle plots, the spectra obtained during the initial exposure stage exhibit one broad maximum, whereas an additional maximum or shoulder becomes distinguishable after 12 h, indicating the emergence of a second relaxation contribution. In addition, a line segment with a slope close to 45° is observed in the low-frequency region of the Nyquist plots. This feature is consistent with a significant contribution from diffusion or mass transport. However, a 45° response alone is not sufficient to demonstrate that the entire electrode reaction is controlled exclusively by mass diffusion rather than charge transfer [23,24]. These directly observed changes in the Nyquist and Bode responses provide the objective experimental basis for selecting the equivalent circuits used at different exposure stages.

**Figure 6:**
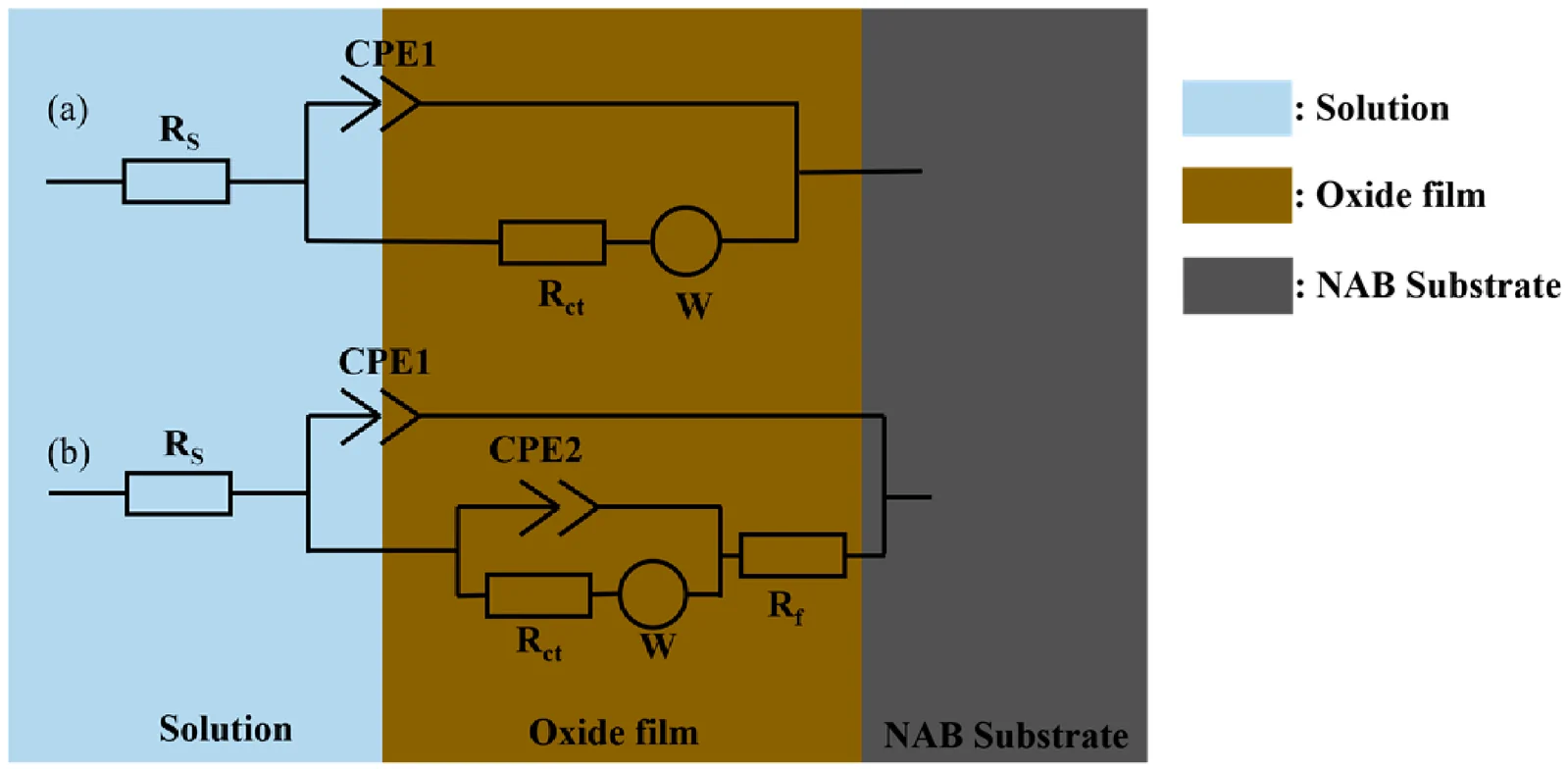
Schematic diagram of electrochemical equivalent circuits: (**a**) initial stage of corrosion; (**b**) late stage of corrosion.

On the basis of these spectral characteristics, the equivalent-circuit models shown in Figure 6 were used to fit the EIS data of the ZCuAl10-4-4-2 copper alloy. The circuit in Figure 6a was used for the initial corrosion stage, for which only one dominant relaxation process can be resolved. The circuit in Figure 6b was used for specimens exposed for 12 h or longer because the measured spectra display an additional relaxation contribution. This selection is also supported by the more pronounced surface changes, the increased accumulation of corrosion products, and the thickening of the corrosion-product film after prolonged exposure. Thus, the more complex circuit was introduced in response to both the measured impedance features and the independent surface observations, rather than being selected solely on the basis of numerical fitting.

In Figure 6, Rs denotes the solution resistance. *R*_ct_ is the charge-transfer resistance associated with the corrosion reaction of the copper alloy; under otherwise comparable conditions and for the same circuit model, a higher *R*_ct_ generally indicates greater resistance to interfacial charge transfer. *R*_f_ is the resistance of the corrosion-product film and is related to the resistance of the film to penetration by the corrosive electrolyte [25]. *CPE*1 and *CPE*2 are constant-phase elements used to describe non-ideal capacitive behavior. In the late-stage circuit, *CPE*1 is mainly associated with the corrosion-product film, whereas *CPE*2 represents the buried alloy/electrolyte interface. *W* is the Warburg impedance element used to represent the diffusion contribution.

The time constants derived from the spectra should be interpreted as distinguishable electrochemical relaxation processes within the measured frequency range, rather than being assumed to correspond one-to-one to the number of independent interfacial reactions [26]. Accordingly, the single relaxation response observed at the initial stage is consistent with an impedance response dominated by the exposed or weakly covered alloy/electrolyte interface, but it does not by itself prove that the electrolyte is in direct contact with every part of the substrate or that only one chemical reaction occurs. After a more developed corrosion-product film forms, two relaxation contributions can be distinguished. The high-frequency contribution is mainly assigned to the non-ideal capacitance and resistance associated with the corrosion-product film, whereas the lower-frequency contribution is attributed primarily to interfacial charge transfer, double-layer charging, and the accompanying mass-transport process beneath the film.

For the late-stage circuit in Figure 6b, the polarization resistance was operationally calculated as the sum of the two fitted finite resistive contributions, *R*_p_ = *R*_f_ + *R*_ct_. This operational definition excludes the solution resistance, the constant-phase elements, and the Warburg element. Because the impedance of a semi-infinite Warburg element increases without bound as the frequency approaches zero, *R*_f_ + *R*_ct_ should be reported explicitly as an operational resistance index rather than as the strict zero-frequency limit of the complete circuit [27,28].

The expression of the equivalent circuit impedance *Z* in Figure 6 is as follows: Figure 6a:

(2) $$z=R_{s}+\frac{1}{\mathrm{CPE}1+\frac{1}{R_{\mathrm{ct}}+W}}$$

Figure 6b:

(3) $$z=R_{s}+\frac{1}{\mathrm{CPE}1+\frac{1}{R_{f}+\frac{1}{\mathrm{CPE}2+\frac{1}{R_{\mathrm{ct}+W}}}}}$$

The equivalent circuit fitting results derived from the EIS of ZCuAl10-4-4-2 copper alloy after different erosion–corrosion exposure times are summarized in Table 5. At the initial corrosion stage, no effective corrosion product film is evident from the EIS response, and the electrolyte remains in direct contact with the alloy substrate. The corrosion process is governed by both charge transfer and mass diffusion at this stage. The disappearance of the diffusion tail in the impedance spectra is regarded as an indicator that the subsequently formed corrosion product film effectively isolates the corrosive medium from the alloy substrate, and the direct interfacial reaction is consequently inhibited [29].

**Figure 7:**
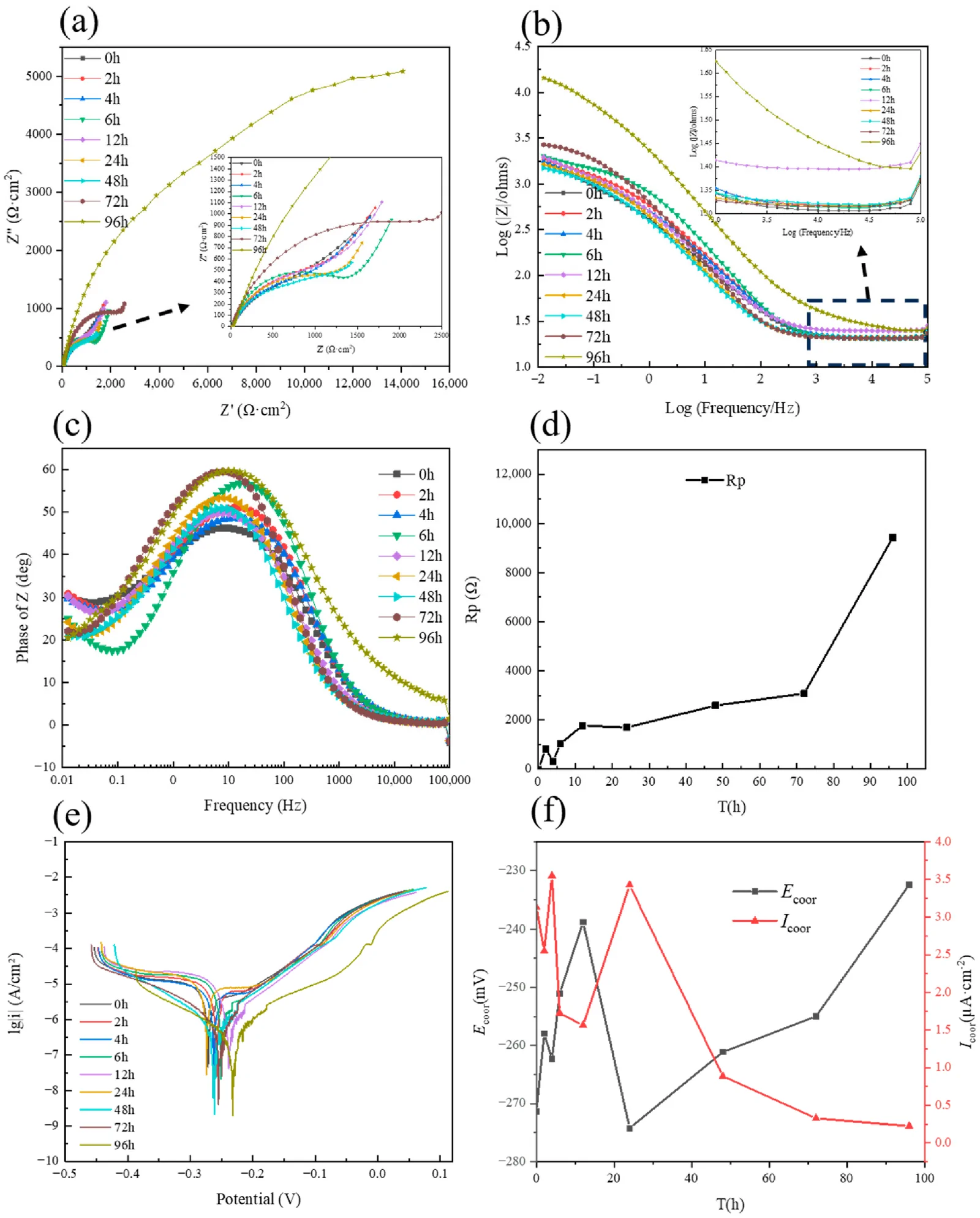
Electrochemical diagrams of the ZCuAl10-4-4-2 copper alloy after different durations of erosion–corrosion: (**a**) Nyquist plot; (**b**) Bode magnitude plot; (**c**) Bode-phase angle plot; (**d**) variation in *R*_p_ values with erosion–corrosion time; (**e**) potentiodynamic polarization curves; (**f**) curves showing the variation in corrosion potential (*E*_corr_) and free corrosion current density (*I*_corr_) with corrosion time. The arrows in (**a**,**b**) indicate magnified views (insets).

Electrochemical impedance measurements were performed on ZCuAl10-4-4-2 copper alloy specimens subjected to different erosion–corrosion exposure periods. As shown in Figure 7a, the diameter of the capacitive arc generally increases with increasing exposure time, indicating a progressive change in the interfacial electrochemical response. Yuan et al. [30] reported that a larger capacitive arc in a Nyquist plot is generally associated with greater resistance to interfacial corrosion reactions. For specimens exposed for 72 h or longer, the low-frequency diffusion tail becomes less pronounced, suggesting a reduced contribution of mass transport to the overall impedance response. This change may be associated with the gradual development of a corrosion-product layer on the alloy surface; however, the protective nature of this layer cannot be established solely from the diffusion-tail characteristics and should be evaluated together with the other electrochemical and surface-characterization results. To quantitatively compare the corrosion resistance of the specimens, the polarization resistance, (*R*_p_), was calculated from the fitted equivalent-circuit parameters. The variation in (*R*_p_), rather than being used to represent the directly measured capacitive-arc size, provides quantitative support for the corrosion-resistance trend observed in the Nyquist plots, with a higher (*R*_p_) indicating greater resistance to interfacial corrosion processes.

The evolution of the *R*_p_ value with erosion–corrosion time is illustrated in Figure 7d. A rapid increase in *R*_p_ is detected within the initial 2 h of erosion–corrosion, suggesting the prompt formation of a primary corrosion product film via the reaction between the alloy substrate and the corrosive medium. A slight reduction in *R*_p_ is observed at 4 h, implying that the formation rate of corrosion products between 2 h and 4 h is exceeded by the erosion-induced removal rate. The *R*_p_ value is elevated from 4 h to 12 h, with a minor peak attained at 12 h. Nevertheless, a lower R_p_ value is recorded at 24 h compared with that at 12 h, indicating that the formation rate of the corrosion product film is again surpassed by erosive elimination, or partial spallation of the film is induced by dynamic erosion between 12 h and 24 h. A gradual increment in the *R*_p_ value is detected with further extension of erosion–corrosion duration beyond 24 h.

The high-frequency impedance magnitude, where the Bode plot exhibits a relatively flat plateau, is primarily associated with *R*_s_. Relatively high system impedance is observed at the 12 h mark in the high-frequency impedance magnitude plot (Figure 7b). Subsequently, the system impedance is found to decrease initially and then increase with prolonged erosion time, reaching its maximum value at 96 h. This trend is generally consistent with the evolution of the *R*_p_ value.

#### 3.3.2. Potentiodynamic Polarization Curve Analysis

The corrosion potential (*E*_corr_) is the mixed potential established under open-circuit conditions, at which the anodic and cathodic partial currents are balanced. It reflects changes in the electrochemical nobility and surface state of the copper alloy in seawater. However, because *E*_corr_ is governed by both anodic and cathodic reaction kinetics, it should not be used alone as a quantitative measure of corrosion resistance or corrosion rate. Under otherwise identical conditions, a positive shift in *E*_corr_ may indicate surface ennoblement or passivation, but this interpretation should be verified using other electrochemical parameters. The corrosion current density (*I*_corr_), obtained from polarization-curve analysis, is a kinetic parameter associated with uniform corrosion. Under comparable experimental conditions, a higher *I*_corr_ indicates a higher uniform corrosion rate and can be converted into a mass-loss or penetration rate according to Faraday’s law [31,32].

In the present work, the corrosion potential (*E*_corr_) and free corrosion current density are obtained via curve fitting based on the polarization curves acquired from specimens exposed to varied erosion–corrosion durations. The potentiodynamic polarization curves of the ZCuAl10-4-4-2 copper alloy following various erosion–corrosion periods are displayed in Figure 7e. The fitted values of the corrosion potential (*E*_corr_) and free corrosion current density, as well as their evolutionary trends, are listed in Table 6 and illustrated in Figure 7f, respectively.

Table 6 and Figure 7f present the data and evolutionary trends of the ZCuAl10-4-4-2 copper alloy during a 96-h dynamic seawater erosion–corrosion process. The corrosion potential (*E*_corr_) exhibits a fluctuating upward trend, shifting positively from −271.408 mV at 0 h to −232.398 mV at 96 h. Two distinct peaks appear at 2 h and 12 h, while pronounced decreases are observed at 4 h and 24 h, with 24 h reaching the most negative value of -274.249 mV. Conversely, the free corrosion current density (*I*_corr_) decreases overall from 3.132 to 0.223 μA·cm^−2^, but exhibits localized spikes at 4 h and 24 h. A clear negative correlation is observed between *E*_corr_ and *I*_corr_. The data confirm the following dynamic evolution mechanism: at 4 h and 24 h, high-speed dynamic seawater scouring leads to a decrease in the protective efficacy of the corrosion product film, resulting in a decrease in *E*_corr_ and an increase in *I*_corr_. In contrast, prolonged exposure (48–96 h) results in the formation of a protective film, enhancing the corrosion resistance of the alloy, as evidenced by the most positive *E*_corr_ and the lowest *I*_corr_ at 96 h.

### 3.4. Corrosion Weight Loss Test Results 

**Figure 8:**
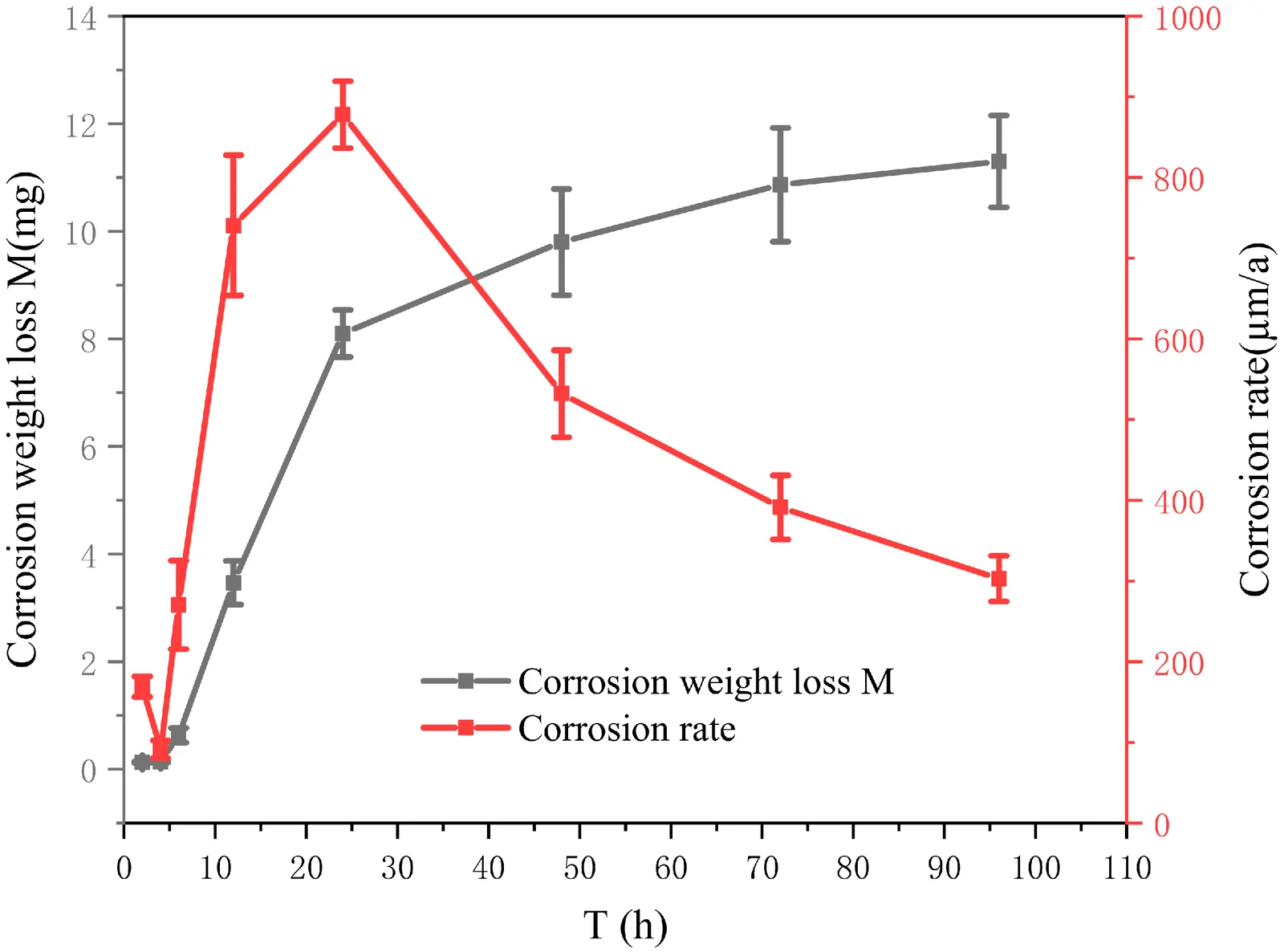
Corrosion weight loss of ZCuAl10-4-4-2 copper alloy.

Corrosion rates are determined on the basis of data acquired from corrosion weight loss measurements. The variation in the corrosion rate of the ZCuAl10-4-4-2 copper alloy with prolonged erosion–corrosion duration is presented in Figure 8, where an initial increase followed by a subsequent decrease is detected, with the maximum corrosion rate attained at 24 h. This phenomenon is ascribed to the reduced polarization resistance and corrosion potential (*E*_corr_), as well as the elevated free corrosion current density (*I*_corr_) recorded at 24 h.

As illustrated in Figure 7d,f and Figure 8, a positive correlation is identified between the evolutionary trend of the *R*_p_ and that of the corrosion potential (*E*_corr_). Similarly, a positive correlation is observed between the evolutionary trend of the free corrosion current density (*I*_corr_) and the corrosion rate. In contrast, a negative correlation is determined between the corrosion potential (*E*_corr_) and the free corrosion current density.

At the initial corrosion stage (2 h), a pronounced increment in both the *R*_p_ value and corrosion potential (*E*_corr_) is observed, suggestive of the primary formation of a corrosion product film; nevertheless, no significant elevation in the corrosion rate is detected at this stage. With the extension of erosion–corrosion time, the corrosion weight loss of the ZCuAl10-4-4-2 copper alloy is determined to exhibit a trend of rapid initial accumulation followed by a gradual slowdown.

Within the 4–12 h erosion–corrosion interval, a significant increase in the corrosion rate is detected, indicating the rapid formation of the corrosion product film during this stage. Similarly, the rapid formation of the corrosion product film within this interval also leads to increases in the *R*_p_ value and corrosion potential (*E*_corr_).

The maximum average corrosion rate is attained at 24 h, accompanied by relatively low values of the corrosion potential (*E*_corr_) and *R*_p_. A degradation in corrosion resistance is inferred, which is ascribed to the spallation or erosive elimination of the corrosion product film induced by high-speed dynamic seawater scouring.

During the erosion–corrosion period of 24–96 h, a significant reduction in the corrosion rate is observed, signifying that the corrosion product film starts to exert effective protective effects on the copper alloy substrate.

## 4. Discussion

**Figure 9:**
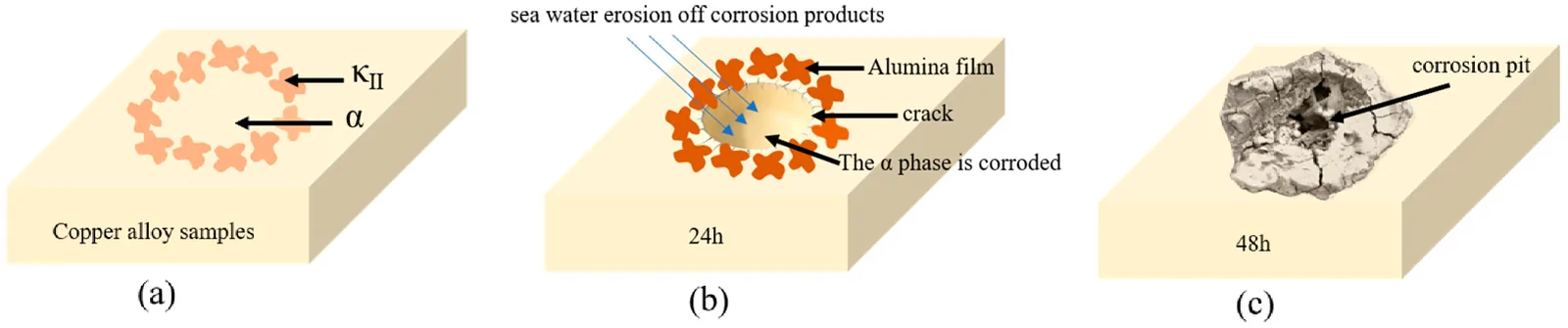
Erosion–corrosion model of ZCuAl10-4-4-2 copper alloy. (**a**) Crack nucleation at 4 h; (**b**) Crack propagation at 24 h; (**c**) Pitting corrosion formation at 48 h.

Simulated seawater erosion–corrosion tests were conducted on the ZCuAl10-4-4-2 copper alloy using a specialized erosion–corrosion testing machine. The phase-selective corrosion characteristics, corrosion kinetics, and damage evolution of the alloy were systematically investigated using SEM, SKPFM, electrochemical measurements, and corrosion weight loss tests. In addition, a comprehensive erosion–corrosion model was established for the target alloy.The erosion–corrosion process of the ZCuAl10-4-4-2 copper alloy is classified into three sequential stages, namely crack nucleation at 4 h (Figure 9a), crack propagation at 24 h (Figure 9b), and pitting corrosion formation at 48 h (Figure 9c). The maximum Volta potential difference is detected between the α phase and the κ_II_ phase, and a lower electrochemical potential is exhibited by the α phase. Consequently, preferential corrosion of the α phase is induced, and microcrack initiation is readily triggered at the κ_II_/α-phase boundaries.

Under the scouring action of high-speed dynamic seawater, a higher corrosion rate is demonstrated by the α phase, while a significantly retarded corrosion rate is observed for the κ phase owing to the protective effect rendered by the surface aluminum oxide film. For erosion–corrosion durations shorter than 24 h, the corrosion products deposited on the specimen surface are susceptible to spallation induced by seawater scouring, which results in intensified corrosion weight loss and degraded corrosion resistance within this period.

When the exposure time exceeds 24 h, a gradual reduction in the corrosion rate is detected for the copper alloy specimen, accompanied by a continuous incremental trend in corrosion resistance. This phenomenon verifies that the oxide film formed on the specimen surface at this stage exerts a stable and effective protective effect.

## 5. Conclusions

Based on the experimental results and corresponding analyses, the key conclusions are summarized as follows:

1. Phase-selective corrosion was identified for the ZCuAl10-4-4-2 copper alloy under simulated seawater erosion–corrosion conditions. The α phase exhibited a lower electrochemical potential compared to the κ phase, thereby rendering the κ/α-phase interfaces susceptible to corrosive attack and conducive to microcrack nucleation. A dense distribution of the κ_II_ phase was detected around these microcracks, and aluminum oxides were confirmed as the dominant corrosion products. Distinct microcracks were observed on the alloy surface following 24 h of erosion–corrosion, while macroscopic pits with a diameter of approximately 100 μm were formed at 48 h of exposure.
2. The evolution of corrosion resistance of the ZCuAl10-4-4-2 copper alloy was characterized under simulated seawater erosion–corrosion conditions. An enhancement in corrosion resistance was observed during the initial 0–2 h of erosion–corrosion. A reduction in corrosion resistance was noted between 2 h and 4 h, which is attributed to the formation rate of corrosion products being lower than their erosion-induced removal rate. Synchronous increases in both corrosion resistance and corrosion rate were observed between 4 h and 12 h, indicating the rapid growth of the corrosion product film. A peak corrosion rate was achieved at 24 h, accompanied by a decline in the protective effectiveness of the corrosion product film. A stable, protective corrosion product film was formed on the alloy surface when the erosion–corrosion duration exceeded 24 h.
3. The corrosion rate of the ZCuAl10-4-4-2 copper alloy was found to first increase and then decrease with increasing erosion–corrosion time, with the maximum corrosion rate recorded at 24 h.

## Figures and Tables

**Table 1:** Chemical composition of nickel aluminum bronze (wt.%).

Cu	Al	Mn	Zn	Fe	Ni	Sn	Pb
77–82	7.0–11.0	0.5–4.0	≤1.0	2.0–6.0	3.0–6.0	≤1.0	≤1.0

**Table 2:** Chemical composition of copper alloy ZCuAl10-4-4-2 (wt.%).

Cu	Al	Mn	Zn	Fe	Ni	Sn	Pb
balance	8.5–10.0	0.8–2.5	≤1.0	3.5–5.5	3.5–5.5	≤1.0	≤1.0

**Table 3:** Composition of artificial seawater.

Compounds	Weight/(g·L^−1^)	Compounds	Weight/(g·L^−1^)
NaCl	24.530	NaHCO_3_	0.201
MgCl_2_	5.200	KBr	0.101
Na_2_SO_4_	4.090	H_3_BO_3_	0.027
CaCl_2_	1.160	SrCl_2_	0.025
KCl	0.695	NaF	0.003

**Table 4:** The content of each phase in ZCuAl10-4-4-2 copper alloy (wt.%).

Phase	Al (wt.%)	Ni (wt.%)	Fe (wt.%)	Mn (wt.%)	Cu (wt.%)
α	8.69	3.31	2.06	2.27	83.67
β′	9.86	3.45	2.53	2.61	81.55
*κ_II_*	16.11	9.51	44.02	3.65	26.71
*κ_III_*	15.43	7.73	31.63	2.83	42.36

**Table 5:** Electrochemical impedance equivalent circuit fitting results of ZCuAl10-4-4-2 copper alloy corrosion for different times.

T	0 h	2 h	4 h	6 h	12 h	24 h	48 h	72 h	96 h
*R*_s_·(Ω·cm^2^)	20.07	20.36	20.42	20.31	24.24	20.72	21.13	20.54	29.82
Q_f_·(F·cm^−2^)	2.522 × 10^−4^	2.751 × 10^−4^	2.607 × 10^−4^	1.624 × 10^−4^	1.111 × 10^−4^	2.507 × 10^−4^	2.954 × 10^−4^	1.904 × 10^−4^	1.044 × 10^−4^
n_f_	0.7570	0.7343	0.7428	0.7940	0.8709	0.8451	0.8114	0.8610	0.7131
*R*_f_·(Ω·cm^2^)	-	-	-	-	32.85	116.2	12.68	365.8	9429
Q_ct_· (F·cm^−2^)	-	-	-	-	4.877 × 10^−4^	4.943 × 10^−4^	6.857 × 10^−4^	3.281 × 10^−4^	1.004 × 10^−4^
n_ct_	-	-	-	-	0.5292	0.4889	0.3306	0.5559	0.4442
*R*_ct_·(Ω·cm^2^)	1.121 × 10^−4^	821.2	318.9	1036	1744	1585	2580	2716	1.199 × 10^−3^
Warburg (S cm^2^S^0.5^)	3372	2233	2519	1509	6984	3369	3824	4548	6765
*R*_p_·(Ω·cm^2^)	1.121 × 10^−4^	821.2	318.9	1036	1777	1701	2593	3082	9429
Chi-Squared	0.00213	0.00401	0.00296	0.00254	0.00131	0.0000751	0.00236	0.00172	0.00430

**Table 6:** Fitting values of natural corrosion potential and natural corrosion current density of the ZCuAl10-4-4-2 copper alloy at different corrosion times.

T (h)	*E*_corr_ (mV)	*I*_corr_ (µA·cm^−2^)
0 h	−271.408	3.132
2 h	−257.970	2.551
4 h	−262.347	3.546
6 h	−251.058	1.725
12 h	−238.772	1.564
24 h	−274.249	3.425
48 h	−261.118	0.886
72 h	−254.975	0.325
96 h	−232.398	0.223

## Data Availability

The original contributions presented in this study are included in the article. Further inquiries can be directed to the corresponding author.

## References

[1] Wharton J., Barik R., Kear G., Wood R., Stokes K., Walsh F. (2005). The corrosion of nickel–aluminium bronze in seawater. Corros. Sci..

[2] Zhang L., Ma A., Yu H., Umoh A., Zheng Y. (2019). Correlation of microstructure with cavitation erosion behaviour of a nickel-aluminum bronze in simulated seawater. Tribol. Int..

[3] Schüssler A., Exner H.E. (1993). The corrosion of nickel-aluminium bronzes in seawater—I. Protective layer formation and the passivation mechanism. Corros. Sci..

[4] Kear G., Barker B.D., Stokes K., Walsh F.C. (2004). Flow influenced electrochemical corrosion of nickel aluminium bronze—Part I. Cathodic polarisation. J. Appl. Electrochem..

[5] Al-Hashem A., Caceres P., Riad W., Shalaby H. (1995). Cavitation corrosion behavior of cast nickel-aluminum bronze in seawater. Corrosion.

[6] Wood R.J.K. (2017). Marine wear and tribocorrosion. Wear.

[7] Song Q., Zheng Y., Ni D., Ma Z. (2015). Studies of the nobility of phases using scanning Kelvin probe mi-croscopy and its relationship to corrosion behaviour of Ni–Al bronze in chloride media. Corros. Sci..

[8] Cui Y., Liu R., Cui Y., Wang F., Liu L. (2025). Effect of temperature gradient on the formed corrosion products films of 70/30 Cu-Ni alloy immersed in 3.5% NaCl solution. Corros. Sci..

[9] Chu H., Yang Z., Cao Y., Zhang W., Xie H., Yuan Y., Wang H., Yue D. (2025). Effect of Chromium on Mechanical Properties and Corrosion Behavior of Copper–Nickel Alloy. Materials.

[10] Wang W., Zhang W., Huang G., Mi X., Huang L. (2024). Effect of in-situ pre-soaking in seawater on the erosion-corrosion properties and micro-mechanism of nickel aluminium bronze alloy. Corros. Sci..

[11] Cao X., Zhang H., Xu Z., Wu H., Qu H., Bian J. (2025). Study on the erosion-corrosion mechanisms of B10 copper-nickel alloy in sand-containing seawater. Eng. Fail. Anal..

[12] Li N., Zhang W., Jin T., Zhao Y., Dai W. Study on Erosion-Corrosion Mechanism of B10 Cu-Ni Alloy in Seawater. Proceedings of the 2018 Prognostics and System Health Management Conference (PHM-Chongqing).

[13] (2021). Standard Practice for Preparation of Substitute Ocean Water.

[14] (2003). Standard Practice for Preparing, Cleaning, and Evaluating Corrosion Test Specimens.

[15] (2023). Standard Practice for Calculation of Corrosion Rates and Related Information from Electrochemical Measurements.

[16] Cottam R., Brandt M., Lawrence J., Waugh D.G. (2015). 20—Laser surface treatment to improve the surface corrosion properties of nickel-aluminum bronze. Laser Surface Engineering.

[17] Nakhaie D., Davoodi A., Imani A. (2014). The role of constituent phases on corrosion initiation of NiAl bronze in acidic media studied by SEM–EDS, AFM and SKPFM. Corros. Sci..

[18] Ding Y., Zhao R., Qin Z., Wu Z., Wang L., Liu L., Lu W. (2019). Evolution of the Corrosion Product Film on Nickel-Aluminum Bronze and Its Corrosion Behavior in 3.5 wt% NaCl Solution. Materials.

[19] Lv Y.T., Guo J.W., Zhang G.S., Cao L.M., Sun X.Y., Qin Z.B., Xia D.H. (2022). Insights into the selective phase corrosion of as cast NiAl bronze alloy: Effect of electrical properties of each phase’s protective film. J. Alloys Compd..

[20] Li L., Qiao Y.X., Zhang L.M., Ma A.L., Ma R.Y., Zheng Y.G. (2023). Understanding the corrosion behavior of nickel–aluminum bronze induced by cavitation corrosion using electrochemical noise: Selective phase corrosion and uniform corrosion. Materials.

[21] Cai X., Yang M.M., Li S.J., Wang C., Qiao Y.X., Zhou J., Xue F. (2025). Comparative analysis of cavitation erosion behavior in wire-arc directed energy deposition and cast nickel-aluminum bronze alloys. Ultrason. Sonochem..

[22] Culpan E.A., Rose G. (1979). Corrosion Behaviour of Cast Nickel Aluminium Bronze in Sea Water. Br. Corros. J..

[23] Lazanas A.C., Prodromidis M.I. (2023). Electrochemical Impedance Spectroscopy—A Tutorial. ACS Meas. Sci. Au.

[24] Wang S., Zhang J., Gharbi O., Vivier V., Gao M., Orazem M.E. (2021). Electrochemical impedance spectroscopy. Nat. Rev. Methods Primers.

[25] Beccaria A.M., Poggi G., Traverso P., Ghiazza M. (1991). A study of the de-alloying of 70Cu-30Ni commercial al-loy in sulphide polluted and unpolluted sea water. Corros. Sci..

[26] Hernández R., Pászti Z., de Melo H., Aoki I. (2010). Chemical characterization and anticorrosion proper-ties of corrosion products formed on pure copper in synthetic rainwater of Rio de Janeiro and São Paulo. Corros. Sci..

[27] Wang W.-Y., Zhang W.-J., Huang G.-J., Mi X.-J. (2025). Synergistic improvement of erosion-corrosion resistance and mechanical properties of nickel aluminium bronze alloy by the addition of Cr. Rare Met..

[28] Tan Y., Xie H., Feng X., Huang S., Zhang W., Peng L., Wang W., Zhao Y. (2024). Effect of Al/Ni Ratio on the Microstructure and Properties of Nickel–Aluminum Bronze Alloys. Materials.

[29] Nady H., El-Rabiei M.M., Samy M. (2017). Corrosion behavior and electrochemical properties of carbon steel, commercial pure titanium, copper and copper–aluminum–nickel alloy in 3.5% sodium chloride containing sulfide ions. Egypt. J. Pet..

[30] Yuan S.J., Pehkonen S.O. (2007). Surface characterization and corrosion behavior of 70/30 Cu–Ni alloy in pris-tine and sulfide-containing simulated seawater. Corros. Sci..

[31] Knisz J., Eckert R., Gieg L.M., Koerdt A., Lee J.S., Silva E.R., Skovhus T.L., Stepec B.A.A., Wade S.A. (2023). Microbiologically influenced corrosion—More than just microorganisms. FEMS Microbiol. Rev..

[32] Han J., Gharbi O. (2022). Current state of electrochemical techniques and corrosion rate analysis for next-generation materials. Curr. Opin. Electrochem..

